# Permanent biventricular ICD-implantation in a heart failure second re-do-CABG patient: a case report

**DOI:** 10.1186/1757-1626-2-59

**Published:** 2009-01-15

**Authors:** Omer Dzemali, Nadejda Monsefi, Anton Moritz, Peter Kleine

**Affiliations:** 1 Department of Thoracic & Cardiovascular Surgery, Johann Wolfgang Goethe University, Frankfurt am Main, Germany

## Abstract

Biventricular pacing has been suggested in end-stage heart failure. We present a 59-year-old patient undergoing second re-do CABG (coronary artery bypass graft) and carotid artery endarterectomy. Ejection fraction was 15%, QRS-width 175 ms. Following the carotid and CABG procedure, an implanted single-chamber ICD (implantable cardioverter defibrillator) was upgraded to permanent biventricular DDD pacing by implantation of one epicardial left ventricular and one epicardial atrial electrode. At follow-up two months postoperatively ejection fraction had significantly improved to 45%, the patient underwent stress test with adequate load and reported a good quality of life.

## Introduction

Widening of QRS complexes to more than 150 ms has been defined to be one independent risk factor for cardiac mortality in patients with poor left ventricular function [1]. In these patients resynchronization of the interventricular conduction system by biventricular stimulation leads to improvement of quality of life and NYHA (New York Heart Association) class [2,3]. In open heart operations the acute hemodynamic benefit of biventricular pacing can be used by implantation of temporary epicardial electrodes facilitating weaning from extracorporeal circulation in high risk patients with impaired left ventricular function and improving cardiac output postoperatively [4-6]. Intraoperative implantation of permanent epicardial pacing leads and devices can combine the immediate and the long-term advantages of biventricular stimulation.

## Case presentation

Four weeks prior to the operation a 59-year-old male was admitted to hospital for unstable angina and dyspnea at rest (NYHA III-IV). Following preoperative diagnostics the patient was referred to our hospital for carotid endarterectomy and a simultaneous second re-do CABG procedure after previous surgery 7 and 15 years earlier. Concomitant diseases were diabetes, renal insufficiency and chronic hepatitis. Nine months prior to admission a single-chamber ICD system had been implanted for recurrent ventricular tachycardias. The ECG showed sinus rhythm with AV-block I° and a complete left bundle-branch block with QRS-width of 170 ms. Preoperative echocardiography demonstrated an ischemic cardiomyopathy with inferior akinesia, septum bulging and an ejection fraction of 15%, moderate mitral insufficiency II° and moderate pulmonary hypertension. Positron emission tomography revealed a globally thin and dilated left ventricle with a transmural inferior scar and hibernating myocardium in the remaining segments.

Immediately before initiation of anesthesia an intra-aortic balloon pump was implanted to prevent hemodynamic instability throughout the procedure. The carotid disobliteration was followed by the coronary operation with revascularization of the left anterior descending artery and the right coronary artery using the left internal thoracic and right radial arteries. The circumflex branches were too small for revascularization. Intraoperatively the left ventricle was enlarged with an inferior scar and globally reduced contractility Following release of the aortic cross clamp and completion of the proximal aortic anastomosis, mapping of the lateral and anterior left ventricular wall was performed under echocardiographic control using an epicardial screw-in electrode CapSure Epi 4965 (Medtronic Inc, Minneapolis, MN, USA). Paradox septum motion disappeared with left ventricular stimulation; the most symmetric contraction was achieved by stimulating a segment at the basis of the lateral wall close to the left atrial appendage. The electrode was implanted with a pacing threshold of 1.2 V (Volt) and an R-wave sensing of 11.5 mV (milliVolt). An additional bipolar epicardial electrode Medtronic CapSure Epi 4968 was placed at the lateral wall of the right atrium. The incision at the left upper thorax was reopened; the defibrillation electrode was connected to the new three-chamber-ICD SJM Epic™ (St. Jude Medical Inc, St. Paul, MN, USA). Both epicardial electrodes were transferred into the ICD pocket through the left hemithorax and connected as well.

**Figure 1:**
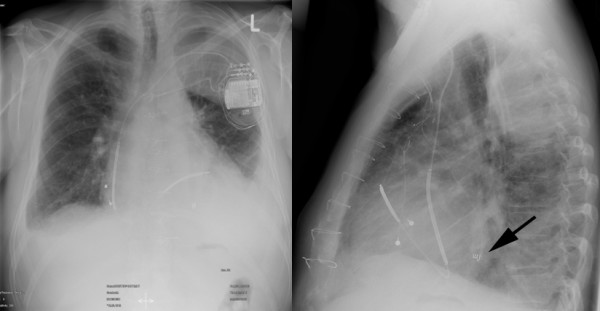
**Postoperative x-ray demonstrating location of the transvenous ICD electrode, the left ventricular epicardial screw-in electrode at the lateral wall (arrow) and the bipolar epicardial lead at the right atrium**.

Weaning from extracorporeal circulation was achieved under continuous DDD biventricular pacing at a rate of 85/min and moderate hemodynamic support (epinephrine 0.42 μg/kg/min). Total bypass time was 142 min. The remaining intra- and early postoperative course was uneventful except for a re-sternotomy for diffuse bleeding. The hemodynamic condition improved with a normal cardiac output throughout the intensive care stay. On the second postoperative day inotropic support was completely weaned, the intra-aortic balloon pump was removed and the patient was extubated. On the 7^th ^day he developed right-sided pneumonia and was artificially ventilated for another three days, but recovered well and could be mobilized during the following week. He was transferred to a rehabilitation centre on the 18^th ^day with no signs of congestive heart failure (Figure 1).

Two and eight months after the operation, the patient presented for a follow-up visit in good condition. He did not complain of angina or dyspnea anymore (NYHA I), his stress test up to 90 W was uneventful except for physical fatigue, no ECG changes were seen under continuous DDD pacing, QRS-width was 145 ms. Echocardiography showed an unchanged inferior akinesia, but a normal septum motion with an improved global left ventricular function. Ejection fraction was 45%, mitral insufficiency was reduced to 0-I°.

## Discussion

Various studies have demonstrated efficacy of biventricular pacing in advanced heart failure [2,3,7]. The acute hemodynamic improvement was also observed in the immediate postoperative course in patients undergoing open heart surgery with impaired left ventricular function [4-6]. Compared to conventional methods for treatment of perioperative heart failure like intra-aortic balloon implantation or administration of high doses of inotropic support, biventricular pacing can be easily initiated and is accompanied with almost no side effects. In our case we combined the short and long term effects of resynchronization therapy in a patient undergoing a technically challenging operation with severely depressed left ventricular contractility and regional wall motion abnormalities. This simultaneous approach has to be considered superior to a two stage operation, as the immediately improved left ventricular function can already be used for weaning from extracorporeal circulation. This was uncomplicated in our patient with only moderate inotropic support, even postoperative complications like bleeding and pneumonia, which was probably related to the long preoperative hospitalization, did not lead to hemodynamic instability.

Compared to a transvenous approach, epicardial electrode implantation allows mapping of the whole left ventricular wall with optimization of the electrode placement and adds only a short period of operation time. The positive effect of biventricular stimulation on regional wall contractility can be monitored by transesophageal echocardiography. In our case inferior wall and interventricular septum motion abnormality were demonstrated initially, the latter a phenomenon which is very common in open heart surgery not only in patients with reduced ventricular function and left bundle-branch block. Biventricular pacing led to immediate normalization of this septum bulging.

During early follow-up the patient improved dramatically regarding his NYHA classification due to a combination of myocardial revascularization and permanent biventricular pacing. His improved quality of life and his almost normal stress test could be related to an increased ejection fraction and decreased mitral insufficiency.

In summary, we suggest a generous consideration of permanent biventricular stimulation during open heart surgery in patients with severely impaired left ventricular function and widened QRS-complexes. The technique adds only little extra time, allows optimal lead location and monitoring by transesophageal echocardiography. It facilitates the early postoperative phase and adds extra benefit to the quality of life in these high risk patients. Long-term effects and impact on mortality during follow-up will have to be investigated.

## Competing interests

The authors declare that they have no competing interests.

## Authors' contributions

OD and PK performed the operation.

NM analyzed the patient data.

AM did the final proof-reading of the manuscript.

## Consent

Written informed consent was obtained from the patient for publication of this case report and accompanying images. A copy of the written consent is available for review by the Editor-in-Chief of this journal.
